# Short-Term Efficacy and Safety of Oral and Nasal Corticosteroids in COVID-19 Patients with Olfactory Dysfunction: A European Multicenter Study

**DOI:** 10.3390/pathogens10060698

**Published:** 2021-06-04

**Authors:** Sven Saussez, Luigi Angelo Vaira, Carlos M. Chiesa-Estomba, Serge-D. Le Bon, Mihaela Horoi, Giovanna Deiana, Marzia Petrocelli, Philippe Boelpaep, Giovanni Salzano, Mohamad Khalife, Stephane Hans, Giacomo De Riu, Claire Hopkins, Jerome R. Lechien

**Affiliations:** 1 COVID-19 Task Force of the Young-Otolaryngologists of the International Federations of Oto-Rhino-Laryngological Societies (YO-IFOS), 75000 Paris, France; lavaira@uniss.it (L.A.V.); chiesaestomba86@gmail.com (C.M.C.-E.); serge-daniel.lebon@stpierre-bru.be (S.-D.L.B.); mihaela_horoi@stpierre-bru.be (M.H.); mohamad.khalife@epicura.be (M.K.); prhans.foch@gmail.com (S.H.); gderiu@uniss.it (G.D.R.); jerome.lechien@umons.ac.be (J.R.L.); 2 Department of Human and Experimental Oncology, Faculty of Medicine UMONS Research Institute for Health Sciences and Technology, University of Mons (UMons), 7000 Mons, Belgium; philippe.boelpaep@umons.ac.be; 3 Department of Otorhinolaryngology and Head and Neck Surgery, CHU de Bruxelles, CHU Saint-Pierre, School of Medicine, Université Libre de Bruxelles, 1000 Brussels, Belgium; 4 Department of Otolaryngology-Head & Neck Surgery, EpiCURA Hospital, 7331 Baudour, Belgium; 5 Maxillofacial Surgery Operative Unit, Department of Medical, Surgical and Experimental Sciences, University of Sassari, 07100 Sassari, Italy; giovannisalzanomd@gmail.com; 6 Biomedical Science Department, PhD School of Biomedical Science, University of Sassari, 07100 Sassari, Italy; 7 Department of Otorhinolaryngology-Head & Neck Surgery, Hospital Universitario Donostia, 20009 San Sebastian, Spain; 8 Direction, Hygiene and Hospital Infection Control Operative Unit, Department of Medical, Surgical and Experimental Sciences, University of Sassari, 07100 Sassari, Italy; giovanna.deiana90@gmail.com; 9 Maxillofacial Surgery Operative Unit, Bellaria and Maggiore Hospital, AUSL, 40100 Bologna, Italy; marzia.petrocelli@ausl.bologna.it; 10 Maxillofacial Surgery Operative Unit, University Hospital of Naples “Federico II”, 80100 Naples, Italy; 11 Department of Otolaryngology-Head & Neck Surgery, Foch Hospital, School of Medicine, UFR Simone Veil, Université Versailles Saint-Quentin-en-Yvelines (Paris Saclay University), 92150 Paris, France; 12 Guy’s and St Thomas NHS Foundation Trust, London E17AF, UK; clairehopkins@yahoo.com; 13 British Rhinological Society (President), London E17AF, UK

**Keywords:** COVID-19, SARS-CoV-2, anosmia, olfactory, smell, treatment

## Abstract

*Background*: The objective of this study was to investigate the efficacy and safety of early administration of oral corticosteroids (OC) or nasal corticosteroids (NC) as an add-on to olfactory training (OT) versus OT alone in patients with olfactory dysfunction (OD) related to coronavirus disease 2019 (COVID-19). *Methods*: Patients with a positive diagnosis of COVID-19 and OD were prospectively recruited from March 22 to December 15, 2020 from 4 European hospitals. Patients had confirmed OD on psychophysical testing. All patients undertook OT, with add-on 10 days of OC (group 1: OC + OT), or 1 month of NC (group 2: NC + OT) or olfactory training alone (group 3: OT). Olfactory evaluations (Sniffin’Sticks tests) were carried out at the time of inclusion, 1 and 2 months after the start of the therapeutic course. *Results*: A total of 152 hyposmic or anosmic patients completed the study. Group 1, 2 and 3 included 59, 22 and 71 patients, respectively and all patient groups were comparable regarding baseline Sniffin’Sticks tests. The median Sniffin’Sticks test values significantly improved from pre- to post-intervention in all groups. The increase of Sniffin’Sticks test values was higher in group 1 (OC + OT) compared with groups 2 and 3 (*p* < 0.001) at one month after treatment but did not remain so at 2 months. Groups 1, 2 and 3, respectively, presented parosmia in 20/71 (28.2%), 9/22 (40.9%) and 42/71 (59.2%) patients. This difference was statistically significant between group 1 and 3 (*p* < 0.001). There were no patients with a worsening of the disease or an increase of the severity of the COVID-19 symptoms. *Conclusions*: The use of OCs in patients with OD related to mild COVID-19 is generally well-tolerated without any case of deterioration of symptoms. OC is associated with greater improvement in psychophysical olfactory evaluations at 1-month post-treatment but there was no difference at 2 months. Parosmia may be reduced following treatment with OC and NC. On the basis of these preliminary results, it is possible to state that considering the 2 months efficacy of OC and NC with respect to the OT alone and the risk-benefit ratio, the benefit to start a specific treatment of COVID-19 related OD cannot be demonstrated and there is a need for a randomised controlled trial to assess this further.

## 1. Introduction

Coronavirus disease 2019 (COVID-19) has affected 123 million people worldwide and resulted in over two and a half million deaths [1]. Since the onset of the pandemic in Europe, special attention has been paid by otolaryngologists to olfactory (OD) and gustatory dysfunctions (GD), which have been found in 50% to 85% of mild forms of the disease in Europe and North America [2,3,4,5,6,7]. However, many geographic differences have been reported and the overall prevalence worldwide is around 46% [8]. The first studies with 6-month follow-up reported that 5% to 11.7% of patients presented severe long-term OD and GD, meaning that we can expect have a high number of patients with disabling disorders in the next few years [9,10,11,12,13]. However, this data must be interpreted with caution as there are significant differences based on the assessment methodology [14] (e.g., objective versus subjective tests) and in no study it is possible to exclude that patients unknowingly presented an OD before COVID-19 [15].

For this reason, therapeutic measures must be implemented to prevent the persistence of these disorders. The optimal therapeutic management of OD and GD related to COVID-19 is not yet well-established. During the first European wave of the pandemic, patients were advised to undertake olfactory training (OT) while the use of oral corticosteroids (OC) was generally restricted because of immunosuppression and delayed viral clearance. Even short-term use of OC may carry a risk of side effects which must be considered both in the context of the acute infection, and over a lifetime of cumulative risk [16]. On the contrary, OT is a complication-free therapeutic measure that acts by stimulating the neuronal plasticity of the olfactory system [17]. However, many otolaryngological conditions attributed to viral infection are commonly treated by OC, which may improve the recovery of nerve function through a reduction of the post-infectious inflammatory reaction [18,19].

The mechanisms underlying the possible efficacy of OC in COVID-19 related OD may involve an anti-inflammatory effect at the post-infectious stage of the disease in the olfactory neuroepithelium. To date, it is increasingly supported that the development of anosmia is related to local inflammatory reaction associated with the destruction of the sustentacular cells [20,21,22,23]. The progressive involvement of basal cells and olfactory neurons would be at the basis of the persistence of OD [22]. It is therefore possible to hypothesize that the earlier this inflammatory process is turned off the more there is the possibility of preventing persistent olfactory disturbances. The first studies regarding the efficacy of OC and NC in the treatment of post-COVID-19 OD have given conflicting results [24,25,26,27] and some groups of authoritative authors advise against the early administration of corticosteroids in COVID-19 patients with OD due to possible side effects and the general tendency of OD to spontaneous recovery [28,29]. However, these recommendations are not yet supported by objective data. To the best of our knowledge the study by Rashid et al. [26] is the only trial published so far that investigated the effectiveness of a specific treatment (i.e., nasal corticosteroids) administered when the infection is still active. However, this study does not analyze the efficacy of OC and the assessment of smell does not include psychophysical tests [30]. Psychophysical tests are capable of quantifying olfactory impairment and can limit any confounds because they are conducted in a controlled environment with standardized procedures. Moreover, they rely on true perception of olfactory stimuli, diminishing response and measurement bias. Moreover, the self-reported olfactory loss is often prone to recall bias [31]. The aim of this study was to investigate the efficacy and safety of the early administration of OC and NC in patients with OD related to COVID-19.

## 2. Materials and Methods

The prospective, observational controlled study was approved by four Institutional Review Boards: CHU Saint-Pierre, Brussels, Belgium (CHUSP200425, CHUSP210119) Epicura, Badour, Belgium (P2020011, P2020047) Sassari University Hospital, Italy (PG-2021-5471), Hospital Universitario Donostia, Spain (CHD011220). The study was performed respecting the STROBE Statement for observational studies. Patients agreed to participate and completed electronic or paper informed consent forms. In Belgian hospitals, the patients recruited between March and October 2020 were treated with OT, while those recruited from November 2020 to December 2020 received OC. In Sassari hospital, patients recruited from March to November 2020 were treated with OT. In the Spanish hospital patients recruited from November 2020 to December 2020, received nasal corticoids for 1 month. Irrespective to the group, patients were invited to perform olfactory training at least twice daily with >3 daily odors (e.g., coffee, perfume, essential oils) for each OT session. From March 22nd 2020 to December 15th, 2020, patients with a confirmed diagnosis of COVID-19 and OD confirmed by psychophysical tests were recruited within 2 weeks of OD onset from four European hospitals. The severe acute respiratory syndrome coronavirus 2 (SARS CoV2) infection was identified through nasal swabs and positive reverse transcriptase polymerase chain reaction (RT-PCR) or serology (IgM and/or IgG).

### 2.1. Epidemiological and Clinical Outcomes

The following epidemiological data were collected through a standardized online questionnaire or medical records: gender, age, smoking and comorbidities. The following comorbidities were evaluated: hypercholesterolemia, reflux, heart, respiratory, kidney, liver, autoimmune diseases, diabetes, neurological, hypertension, allergy, chronic rhinitis, chronic rhinosinusitis, depression, thyroid and skin disorders. The symptoms of patients during the clinical course of the disease were evaluated with the COVID-19 Symptom Index, which is a 100-point clinical instrument evaluating the common COVID-19 symptoms [32]. In addition, nasal symptoms and the potential relationship between nasal complaints and OD were assessed with the French, Italian and Spanish versions of the sinonasal outcome tool-22 (SNOT-22) [33].

### 2.2. Subjective and Psychophysical Olfactory Evaluations

Subjective olfactory and gustatory functions were evaluated with the smell and taste component of the National Health and Nutrition Examination Survey, which was previously used in our studies [3,34]. Psychophysical olfactory assessments were performed with the identification component of Sniffin’Sticks tests (Medisense, Groningen, the Netherlands), which is a validated psychophysical olfactory test using 16 smell pens. Each pen was presented to individual who had to choose the adequate smell between four given options. The final score ranges from 0 (none correctly identified) to 16 (all correctly identified) Normative values established normosmia as a score ranging between 12–16, hyposmia between 9–11, and anosmia between 0–8 [35,36]. Baseline clinical and olfactory evaluations were performed within the first 2 weeks after OD onset and repeated 1 and 2 months later.

The inclusion criteria consisted of adults with COVID-19 related OD with onset within the previous two weeks and a Sniffin’Sticks test score <11 (anosmia or hyposmia). Patients with the following criteria were excluded: normosmia at the psychophysical evaluation, history of OD, chronic or self-reported acute rhinosinusitis (regarding EPOS guidelines) at the time of evaluation, dementia, or other conditions associated with an inability to complete the evaluations.

### 2.3. Therapeutic Course

Three groups of patients with OD were prospectively treated according to local protocol. Group 1 consisted of patients who received 10 days of OC (methylprednisolone 0.5 mg/kg/day) and OT (Group 1: OC + OT). Group 2 included patients receiving 1 month of nasal corticosteroids (mometasone furoate spray, two sprays in each nostril once daily) and OT (Group 2: NC + OT). Groups 1 and 2 received their treatment 1 to 2 weeks after the onset of COVID-19 symptoms. Group 3 consisted of patients receiving olfactory training alone (Group 3: OT) during the follow-up (depending on recovery). Treatment allocation was determined in part by participating centres. Treatment was determined by hospitals. To assess the safety and subjective efficacy of OC, patients of group 1 were asked to fulfill an online survey evaluating the adverse effects of oral medication and all groups reported on frequency of performing OT prior to the investigator consultation. An online questionnaire evaluating parosmia and phantosmia was completed by participants in March 2021. The investigators met the patients at 1- and 2-months post-treatment to perform the psychophysical evaluations. To investigate the potential impact of OC on the production of anti-SARS-CoV-2 IgG, patients of group 1 and group 3 underwent serological evaluation at 2-months post-intervention.

### 2.4. Statistical Analysis

Statistical analyses were performed using the SPSS for Windows version 22.0 software (IBM Corp, Armonk, NY, USA). According to the type of outcomes, the following tests were used to compare severity groups: Kruskal-Wallis, Fisher’s exact test and Mann-Whitney U test. The pre- to post-intervention changes in Sniffin’Sticks tests were evaluated within and between the groups by analysis of variance (ANOVA) for repeated or independent measures with Tukey’s post-hoc test. Multivariate analysis was used to study the associations between outcomes.

## 3. Results

**Figure 1:**
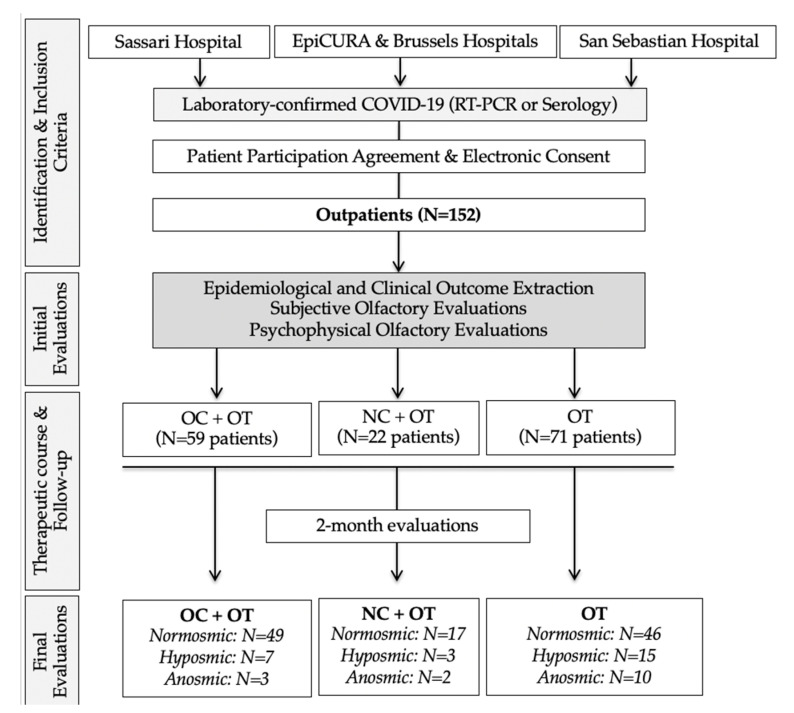
STROBE chart flow. This chart flow was developed according to the STROBE Statements for controlled studies. In EpiCURA and Brussels Hospitals, patients received OT in a first period time and OC in a second period time. Spanish, Italian and Belgian patients were comparable regarding epidemiological and olfactory outcomes at baseline. *Abbreviations*: COVID-19 = coronavirus disease 2019; OC = oral corticosteroids; NC = nasal corticosteroids; OT = olfactory training; RT-PCR = reverse transcription polymerase chain reaction.

A total of 152 patients with OD completed the evaluations (Figure 1). An additional 42 patients self-reported olfactory dysfunction but were normosmic at the psychophysical evaluations and were excluded from the study. The epidemiological and clinical features of patient groups are reported in Table 1.

Fifty-nine patients received OC (group 1). There were 22 and 71 patients in group 2 (NC+ OT) and group 3 (OT), respectively (Table 1). The groups were comparable regarding gender proportion (*p* = 0.612) and age (*p* = 0.236). All patients were classified as mild COVID-19 patients according to the WHO classification [37] and were therefore home-managed. No patient was hospitalized throughout the study period. The most prevalent comorbidities were reflux disease (i.e., gastroesophageal and laryngopharyngeal reflux), hypothyroidism and hypertension. Cough, headache, fever, myalgia and fatigue were the most prevalent symptoms (Table 1). The otolaryngological symptom severity and frequency are reported in Table 2.

**Figure 2:**
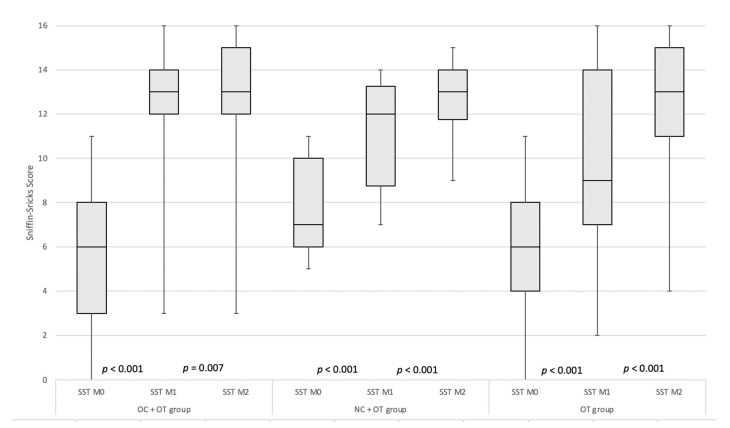
Pre- to Post-intervention evolutions of Psychophysical olfactory evaluations of Groups. The Sniffin’s Sticks test values significantly increased from pre- to post-intervention in all groups. Abbreviations: OC = oral corticosteroids; NC = nasal corticosteroids; OT = olfactory training; SST = Sniffin’Sticks test; M0: Moment 0 (baseline); M1: Moment 1 (30 days); M2: Moment 2 (60 days). The shaded rectangle identifies the IQR around the median, which corresponds to the horizontal line within the rectangle. The error bars identify the maximum and minimum values.

At baseline, the mean SNOT-22 scores of groups did not differ between the groups (*p* = 0.461). The median (interquartile range-IQR) values of Sniffin’Sticks tests were 6 (IQR 5), 7 (IQR 5) and 6 (IQR 5) in groups 1, 2 and 3, respectively. There were no significant differences across groups about the baseline psychophysical olfactory evaluations (*p* = 0.081). Considering all patients, OD developed before (N = 30; 19.7%), during (N = 50; 32.9%) or after (N = 72; 47.4%) the other symptoms (Table 1). Seventy-five patients (49.3%) self-reported taste disorder at baseline. In group 1 (OC + OT), the median Sniffin’Sticks tests increased from 6 (IQR 5) to 13 (IQR 2) and 13 (IQR 3) from pre- to 1-and 2 months post-intervention (Figure 2).

In group 2, the median Sniffin’Sticks tests increased from 7 (IQR 1) to 12 (IQR 4.5) and 13 (IQR 2.25) from pre- to 1 and 2 -months post-intervention (*p* < 0.001) (Figure 2). In patients who only followed OT (group 3), the median Sniffin’Sticks tests increased from 6 (IQR 4) to 9 (IQR 7) and 13 (IQR 4) (*p* < 0.001) (Figure 2).

**Figure 3:**
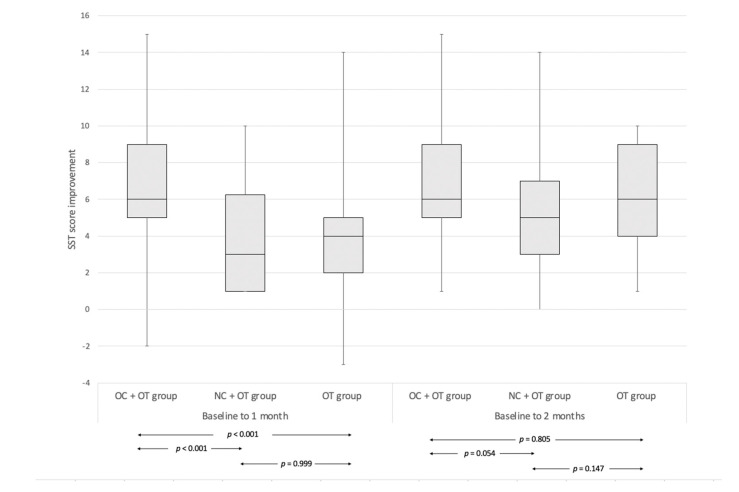
Median olfactory score improvement in groups between baseline and 1 or 2 months. The median olfactory score improvement between baseline and 1-month post-treatment of group OC + OT (6, IQR 4) was significantly higher compared with group NC + OT (3, IQR 5.25; *p* < 0.001) and group OT (4, IQR 3; *p* < 0.001). There were no significant differences between score improvements between groups NC + OT and OT (*p* = 0.999). The median olfactory score improvement between baseline and 2 months post-treatment did not significantly differ between any group. Abbreviations: OC = oral corticosteroids; NC = nasal corticosteroids; OT = olfactory training; SST = Sniffin’Sticks test. The shaded rectangle identifies the IQR around the median, which corresponds to the horizontal line within the rectangle. The error bars identify the maximum and minimum values.

The median olfactory score improvement between baseline and 1-month post-treatment of group OC + OT (6, IQR 4) was significantly higher compared with group NC + OT (3, IQR 5.25; *p* < 0.001) and group OT (4, IQR 3; *p* < 0.001). There were no significant differences between score improvements between groups NC + OT and OT (*p* = 0.999) (Figure 3).

**Figure 4:**
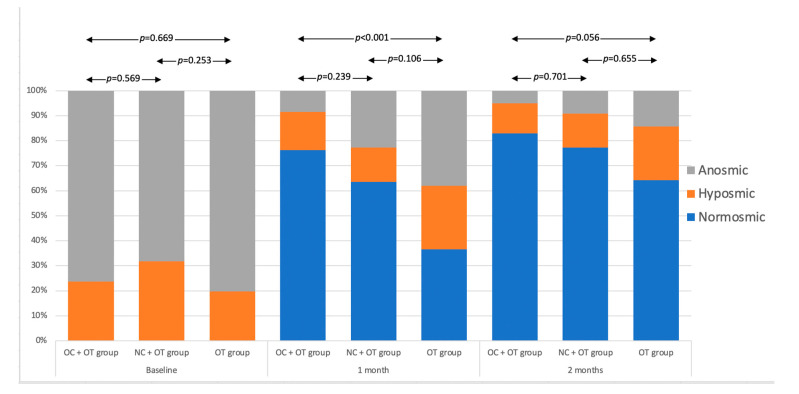
Proportion of Anosmic, Hyposmic and Normosmic Patients 1 and 2 months post-intervention in Groups. The determination of anosmia, hyposmia or normosmia was performed through the identification parts of the Sniffin’Sticks test. The score ranges from 0 (no olfaction) to 16 (perfect olfaction). Normosmia is a score between 12–16. Hyposmia consists of a score ranging from 9 to 11 and anosmia is defined with a score < 9 [35,36].

The median olfactory score improvement between baseline and 2 months post-treatment did not significantly differ between the groups. (Figure 3). The post-intervention proportions of anosmic, hyposmic and normosmic in all groups are available in Figure 4.

**Figure 5:**
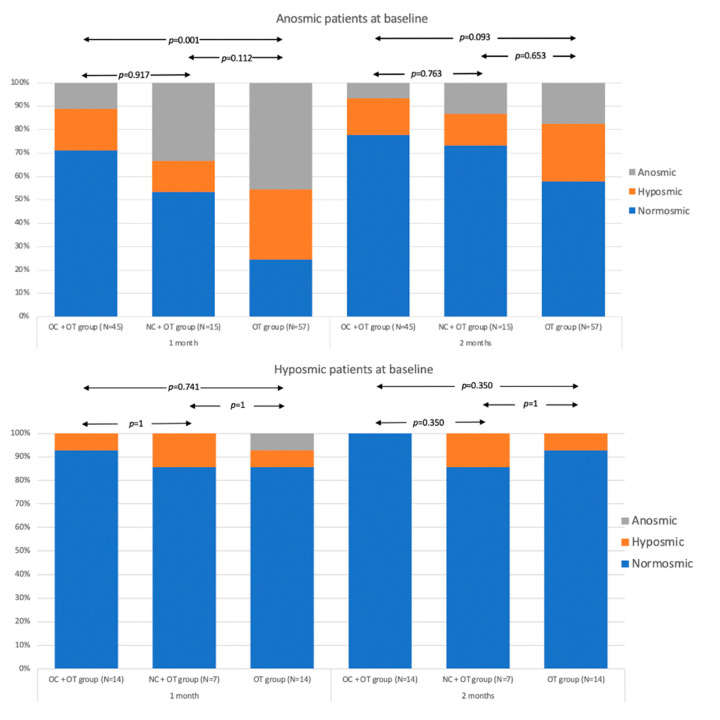
Proportion of Anosmic, Hyposmic and Normosmic Patients 1 and 2 months post-intervention in Sub-groups of patients selected on the basis of OD at baseline. The determination of anosmia, hyposmia or normosmia was perFigure 0. (perfect olfaction). Normosmia is a score between 12–16. Hyposmia consists of a score ranging from 9 to 11 and anosmia is defined with a score < 9 [35,36].

At baseline, there were no significant differences between groups in the proportion of normosmic, hyposmic, and anosmic patients. The proportion of normosmics at 1 month was significantly higher in group 1 (OC + OT) compared with group 3 (OT; *p* < 0.001), the comparison of group 1 to 2 or of group 2 to group 3 was not significant. At 2 months there were no significant differences between the groups. The proportion analysis was also conducted in patient subgroups selected by clinical dysfunction at baseline (Figure 5).

Considering only the hyposmic patients, the differences between the therapy groups were always not-significant, both at 1 month and at 2 months. In patients who were anosmic at baseline, a significantly higher frequency of OD was found in group 3 compared to group 1 at 1-month. This difference was also detected at 2-months but was not-significant at this end-point.

Interestingly, groups 1, 2 and 3 respectively reported having parosmia in 16/59 (27.2%), 9/22 (40.9%) and 42/71 (59.2%) patients. This difference was statistically significant between group 1 and 3 (*p* < 0.001) while it was not between groups 2 and 3 (*p* = 0.149). The mean time between onset and data collection in this regard was 18.4 weeks for group 1, 17.5 weeks for group 2 and 20.2 weeks for group 3.

The adverse effects related to the OC intake were reported in Table 3. The most prevalent adverse effects consisted of insomnia, headache and hypertension. There were no patients with a worsening of the disease or an increase of the severity of the COVID-19 symptoms. At 2-month post-intervention, the anti-SARS-CoV-2 IgG were detected in 54 patients receiving OC + OT (91.5%), while they were detected in 67 patients (94.4%) of the control group (group 3). The use of OC was therefore not associated with a lower proportion of positive serology at 2-months.

## 4. Discussion

The use of oral corticosteroids in COVID-19 remains controversial regarding the hypothetical risk of worsening of the disease through an immunosuppression process or delayed viral clearance, and the risk of side effects. As a result, some scientific societies advised caution with regard to use OC in patients with OD related to COVID-19 [30,31]. These consensus statements add to the evidence base that shows high rates of recovery in all groups regardless of intervention.

The potential benefits of adding OC to OT in COVID-19 patients with olfactory loss was recently suggested by two small studies [24,25], which differ from the present report regarding the time of administration of OC, the patient profiles and the number of included individuals. In the study of Vaira et al., nine patients with anosmia or hyposmia lasting from more than 30 days received both nasal and oral corticosteroids in addition to OT while controls performed OT alone [24]. Overall, they observed that patients treated with OC and nasal corticosteroids reported significantly higher improvements of the olfactory scores than the controls at both day 20 and 40 post-treatment. In the study of Le Bon et al., nine patients received OC and OT more than 1 month after the OD onset, while 18 individuals only undertook OT, with treatment initiated on average 5 weeks after onset if OD. They reported better improvement of olfactory scores in OC group compared with controls [25]. In the current study patients received OC or NC at an earlier stage (within 2 weeks) after the onset of OD, and in a larger group of patients. As observed in the study of Varia et al. and Le Bon et al., we observed a significant difference in terms of improvement in identification scores at 1-month post-treatment, but the difference was no longer significant at 2 months. In terms of the proportion of patients achieving normal scores at 2 months, group 1 was significantly better than group 3. Thus, OC appear to accelerate recovery, but long-term follow-up is needed to determine if this translates into meaningful differences beyond 2 months in terms of the proportion of patients achieving normal olfactory function. The faster recovery demonstrated by OC-treated patients is probably related to the effect that corticosteroids have in rapidly resolving inflammation in the olfactory mucosa allowing for faster regeneration of the epithelium. Although more slowly, this process still occurs in most patients, regardless of treatment, as demonstrated by the improvement in olfactory scores that did not differ significantly between study groups at 60 days. However, early administration of NC does not appear to demonstrate the same efficacy as OC in speeding up olfactory recovery. The number of patients included in the NC + OT group is limited and this result should be interpreted with caution. However, it confirms what has been observed from larger studies on the use of NC for the treatment of COVID-19 related OD [26,27]. The effects of NC on the recovery of olfactory function in COVID-19 patients are well documented by two large randomized controlled trials. Abdelalim et al. [27] analysed two groups of 50 patients with persistent post-COVID-10 OD and treated with mometasone furoate or OT alone, without finding significant differences in olfactory recovery at 3 weeks. This same result was confirmed by Rashid et al. [26] in a placebo-controlled trial that included 356 patients with COVID-19 and OD. The patients were divided into 2 groups: a therapy group of 138 subjects who were treated with the instillation of betamethasone inside the nose and a placebo group, consisting of the same number of subjects, treated with 9% NaCl solution. At the end of the 30-day observation period, no significant differences were found between the two groups.

The present study gives some assurance regarding the safety of OC use in mild COVID-19 patients with OD. We did not observe worsening of the disease severity in any patient after the intake of OC, or an associated reduction in IgG. OC was previously used in patients with post-viral anosmia related to influenzae, Epstein-Barr virus or coronavirus [38]. The safety of OC was supported in the systematic analysis of Harless et Liang who, however, specified that there is a lack of high-quality studies investigating the different therapeutic approaches in post-viral anosmia regarding the low prevalence of the disorder prior to the current pandemic [39]. In the present study, patients who received OC reported adverse effects such as insomnia, headache or hypertension. The OC adverse effects are well-known, and their prevalence does not differ from other studies [20].

However, it must be acknowledged that in addition to the risk of side effects associated with short-term use, there is evidence of a risk of cumulative lifetime harm that must be considered.

In this regard, a large clinical trial supported by the Oxford University (UK RECOVERY trial NCT04381936) has reported a positive survival effect of intravenous dexamethasone administration in the treatment of COVID-19. This study showed that a 10-day treatment with low doses of dexamethasone (6 mg/day) reduced the mortality in patients who required oxygen support. Inhaled corticosteroid (IC) administration attenuated ACE2 expression in mice and airway epithelial cell cultures from patients with COPD [40]. Otherwise, IC administration can also impart a number of detrimental effects on innate immunity including suppression of type I interferon leading to increased virus replication. Interestingly, Matsuyama et al. have recently showed that mometasone, but not budesonide, beclomethasone, or fluticasone, exhibit in vitro suppression of SARS-CoV-2 replication to a similar degree as lopinavir [41]. As demonstrated by UK RECOVERY, the timing of OC or NC administration as well as the dose seems to be a crucial point to be addressed in future studies.

Parosmia is highly prevalent in patients at 6 months and has a significant impact on their quality of life [9]. Our results suggest lower rates of parosmia reported in patients receiving oral or nasal steroids (although the difference reached statistical significance for OC only). A tiny effect is suggested by the apparent benefit seen in group 2 receiving NC, but in the absence of significant improvement in other measures. Given the impact of parosmia on quality of life, patients would likely consider treatment that prevents or reduces the risk of parosmia worthwhile even if the same final olfactory outcomes in other regards are achieved without treatment, and this is an important outcome that needs to be further assessed in trials of interventions.

This study has several limitations. First, the allocation process was not randomized, and patients of the OT group came from the first and the second waves, while the OC and NC groups were recruited in the second wave, and in different centers. There may be temporal or center-based effects that drive differences between groups. The recruitment of patients who came from different regions may involve different variants of SARS-COV-2 or differences in the immune/corticosteroid responses.

The evaluation of the efficacy of OC, NC or OT was not blinded. In order to reduce the impact of this bias, all patients were instructed to fulfill an efficacy online questionnaire before the physical consultation with the investigator who only performed the psychophysical olfactory evaluations. Patients were followed to 2 months and therefore it is possible that with longer term follow-up the same end point would have been reached in all patients [42]. For these reasons, it will be essential to replicate the findings in a placebo controlled randomised study, but our preliminary data supports the need for such a study. The University of Pennsylvania smell identification test, an identification test using 40 odorants, or the extended version of the Sniffin’Sticks tests, which assess also the olfactory threshold and discrimination, may have a greater sensitivity in assessing the olfactory function but they were unavailable on the market at the time the study was started and only the identification part of the Sniffin’Sticks tests was present in the centers involved. Moreover, we wished to minimise patient contact at early stages of infection. Furthermore, this study only included ambulatory COVID-19 patients, defined by WHO criteria as mild patients, and therefore results may not be generalizable to patients with more severe staged of COVID-19. Another limitation to note is the absence of a pre-COVID-19 olfactory evaluation. Since approximately 20% of the general population has an OD [15,43], a significant fraction of patients who do not improve may be those who do not have normal olfaction, unrelated to SARS-CoV-2 infection. Finally, our clinical series was likely still too small to detect significant differences between treatment arms when stratified by the severity of olfactory loss. Although there is a trend of a greater proportion of patients achieving normal olfactory function 2 months after OC, this did not achieve statistical significance, possibly due to type 2 error. In contrast it appears that the administration of either oral or topical corticosteroids in hyposmic patients does not achieve significantly better outcomes as all patients in this group appear to improve regardless of treatment received.

## 5. Conclusions

Oral corticosteroid is an interesting potential therapeutic option for patients with OD related to COVID-19. The use of OC was not associated with worsening of the COVID-19 disease severity. The side effects of OC, although affecting a relevant percentage of patients, did not require discontinuation of therapy, were not serious and spontaneously regressed. Some of these side effects (i.e., headache) are typical of COVID-19 and it cannot be excluded that they were related to the disease rather than to corticosteroid therapy. OC treatment appears to lead to faster recovery, with a larger number of patients achieving normal thresholds in association with a possible reduction in the incidence of parosmia. However, there was no difference regarding OD at 2 months between OC, NC and OT alone. Considering these preliminary results, it is possible to state that on the basis of the 2 months efficacy of OC and NC with respect to the OT alone and the risk-benefit ratio, the benefit of starting steroid treatment of COVID-19 related OD cannot be demonstrated. Future randomized-controlled trials are needed to support our results.

## Figures and Tables

**Table 1:** Epidemiological and Clinical Features of Patient Groups.

	Characteristics	OC + OT (59 Patients)	NC + OT (22 Patients)	OT (71 Patients)	*p*-Value
	Age (y − Mean ± SD)	37.1 ± 11.9	42.95 ± 12.66	43.5 ± 14.25	0.236
	Gender (F/M)	35 F (59.3%)	15 F (68.2%)	40 F (56.4%)	0.612
		24 M (40.7%)	7 M (31.8%)	31 M (43.6%)	
	Current Smoker	5 (8.5%)	1 (4.5%)	11 (15.5%)	0.254
	History of seasonal allergy	8 (13.6%)	4 (18.2%)	9 (12.7%)	0.805
Comorbidities	Diabetes	0 (0)	2 (9.1%)	1 (1.4%)	0.11
	Hypercholesterolemia	2 (3.4%)	2 (9.1%)	1 (1.4%)	0.21
	Hypertension	5 (8.5%)	4 (18.2%)	8 (11.3%)	0.467
	Thyroid disorder	4 (6.8%)	2 (9.1%)	7 (9.8%)	0.818
	Renal insufficiency	0 (0)	0 (0)	0 (0)	1
	Liver insufficiency	0 (0)	0 (0)	0 (0)	1
	Respiratory insufficiency	0 (0)	0 (0)	0 (0)	1
	Asthma	2 (3.4%)	1 (4.5%)	4 (5.6%)	0.831
	Reflux	5 (8.5%)	2 (9.1%)	10 (14.1%)	0.567
	Heart disorder	0 (0%)	0 (0)	3 (4.2%)	0.532
	Depression	0 (0%)	0 (0)	3 (4.2%)	0.532
	Neurological disorder	0 (0)	0 (0)	0 (0)	1
	Skin disorder	5 (8.5%)	0 (0)	0 (0)	0.153
	Autoimmune disorder	0 (0)	0 (0)	4 (5.6%)	0.381
General Symptoms (N–%)	Headache	46 (78%)	13 (59%)	53 (74.6%)	0.222
	Cough	43 (72.9%)	10 (45.4%)	56 (78.9%)	0.02
	Myalgia	42 (71.2%)	11 (50%)	40 (56.3%)	0.114
	Dyspnea	21 (35.6%)	4 (18.2%)	24 (33.8%)	0.305
	Fever (>38C)	35 (59.3%)	18 (81.8%)	33 (46.5%)	0.005
	Arthralgia	32 (54.2%)	10 (45.5%)	35 (49.2%)	0.808
	Diarrhea	26 (44.1%)	9 (40.9%)	41 (57.7%)	0.196
	Fatigue	50 (84.7%)	15 (68.2%)	47 (66.2%)	0.05
	Chest pain	21 (35.6%)	4 (18.2%)	36 (50.7%)	0.016
	Abdominal pain	22 (37.3%)	4 (18.2%)	20 (28.1%)	0.217
	Nausea, vomiting	19 (32.2%)	6 (27.3%)	20 (28.1%)	0.852
	Conjunctivitis	6 (10.2%)	1 (4.5%)	15 (21.1%)	0.075
	Urticaria	5 (8.5%)	0 (0)	4 (5.6%)	0.727
Olfact. disorder onset	SNOT-22	34.4 ± 21.2	38.3 ± 19.94	31.37 ± 13.56	0.462
	Before other symptoms	8 (13.5%)	5 (22.7%)	17 (23.9%)	0.065
	During clinical course of the disease	16 (27.1%)	6 (27.3%)	28 (39.4%)	
	After other symptoms	35 (59.3%)	11 (50%)	26 (36.6%)	
	Taste disorder (self-reported)	27 (45.7%)	10 (45.4%)	38 (53.5%)	0.628

*Abbreviations*: y = years; F/M = female/male; OC = oral corticosteroids; NC = nasal corticosteroids; OT = olfactory training; SD = standard deviation; SNOT-22 = sinonasal outcome test-22.

**Table 2:** Otolaryngological Symptom Severity According to Groups.

	Otolaryngological Symptom Severity														
	Absent (0)			Mild (1)			Moderate (2)			Severe (3)			Very Severe (4)		
	OC + OT	NC + OT	OT	OC + OT	NC + OT	OT	OC + OT	NC + OT	OT	OC + OT	NC + OT	OT	OC + OT	NC + OT	OT
	59	22	71	59	22	71	59	22	71	59	22	71	59	22	71
Nasal obstruction	35 (59.3%)	9 (41%)	30 (42.2%)	8 (13.6%)	2 (9.1%)	22 (31%)	9 (15.2%)	3 (13.6%)	10 (14.1%)	4 (6.8%)	5 (22.7%)	8 (11.3%)	3 (5.1%)	2 (9.1%)	1 (1.4%)
Rhinorrhea	27 (45.8%)	14 (63.6%)	45 (63.4%)	14 (23.7%)	2 (9.1%)	6 (8.4%)	10 (16.9%)	2 (9.1%)	53 (74.6%)	5 (8.5%)	1 (4.5%)	0 (0%)	3 (5.1%)	0 (0%)	0 (0%)
Throat pain	27 (45.8%)	15 (68.2%)	30 (42.2%)	21 (35.6%)	4 (18.2%)	14 (19.7%)	6 (10.2%)	1 (4.5%)	6 (8.4%)	3 (5.1%)	2 (9.1%)	5 (7%)	2 (3.4%)	0 (0%)	1 (1.4%)
Postnasal drip	30 (50.8%)	7 (31.8%)	54 (76.1%)	16 (27.1%)	7 (31.8%)	19 (26.8%)	5 (8.5%)	6 (27.3%)	10 (14.1%)	5 (8.5%)	2 (9.1%)	8 (11.3%)	3 (5.1%)	0 (0%)	4 (5.6%)
Face pain/heaviness	42 (71.2%)	20 (90.9%)	54 (76.1%)	7 (11.9%)	0 (0%)	10 (14.1%)	5 (8.5%)	1 (4.5%)	5 (7%)	4 (6.8%)	1 (4.5%)	2 (2.8%)	1 (1.7%)	0 (0%)	0 (0%)
Dysphonia	48 (81.3%)	20 (90.9%)	29 (40.8%)	6 (10.2%)	2 (9.1%)	10 (14.1%)	5 (8.5%)	0 (0%)	5 (7%)	0 (0%)	0 (0%)	2 (2.8%)	0 (0%)	0 (0%)	0 (0%)
Nasal burning	37 (62.7%)	19 (86.4%)	29 (40.8%)	10 (16.9%)	1 (4.5%)	28 (39.4%)	5 (8.5%)	2 (9.1%)	13 (18.3%)	5 (8.5%)	0 (0%)	1 (1.4%)	2 (3.4%)	0 (0%)	0 (0%)

The symptoms of patients during the clinical course of the disease were evaluated with a 5-point scale ranging from 0 (absent) to 4 (very severe symptoms). Abbreviations: OC = oral corticosteroids; NC = nasal corticosteroids; OT = olfactory training.

**Table 3:** Adverse Effects of Oral Corticosteroid Therapy.

Adverse Effects	OC + OT	
	N	%
Insomnia	26	44.1
Headache	9	16.9
Palpitation	8	13.6
Edema	3	5.1
Weight gain	4	6.8
Euphoria	2	3.4
Stomach/abdominal discomfort	2	3.4
Depression	0	
Asthenia	0	
Diarrhea	0	

The patients receiving oral corticosteroids were invited to note any potential adverse effects during their 10-day therapeutic course.

## Data Availability

S Saussez, LA Vaira LA and JR Lechien had full access to all of the data in the study and take responsibility for the integrity of the data and the accuracy of the data analysis.
